# ENVIRONMENTAL FORTITUDE: NEIGHBORHOOD CONTEXT AND COGNITIVE HEALTH

**DOI:** 10.1093/geroni/igae098.0482

**Published:** 2024-12-31

**Authors:** Noah Webster, Kyriakos Markides

**Affiliations:** Chair; Discussant

## Abstract

It is estimated that up to forty percent of Alzheimer’s Disease and Related Dementia (ADRD) cases are preventable. While modifiable risk factors are often linked with neighborhood characteristics, less attention is given to this context in interventions. Further, as prevalence of ADRD rises and racial/ethnic disparities persist, research is needed within groups to guide development of culturally and environmentally specific interventions. This symposium includes four complementary papers focusing on diverse neighborhood factors and their associations with cognitive health among a sample of older Mexican Americans from the Hispanic Established Population for the Epidemiological Study of the Elderly. The papers result from participation in the Michigan Center for Contextual Factors in Alzheimer’s Disease (MCCFAD) 2023 Summer Data Immersion Program at the University of Michigan. MCCFAD is an NIA funded ADRD Resource Center for Minority Aging Research. Milani and colleagues found that limitations in activities of daily living mediate the association between living in a census tract with more highways and worse cognitive health. Clark and colleagues investigate the effect of ethnic enclaves and demonstrate a negative effect on both cognitive and functional decline. Lee and colleagues found that in urban settings, presence of religious organizations is associated with better cognitive health, while more civic organizations is linked to worse. Scambray and colleagues document an adverse effect of living in a disadvantaged neighborhood on cognition among those reporting moderate levels of relationship quality. These papers will be discussed by Kyriakos Markides who will provide an outlook for future research in this area.

